# Axial Length/Corneal Radius Ratio: Association with Refractive State and Role on Myopia Detection Combined with Visual Acuity in Chinese Schoolchildren

**DOI:** 10.1371/journal.pone.0111766

**Published:** 2015-02-18

**Authors:** Xiangui He, Haidong Zou, Lina Lu, Rong Zhao, Huijuan Zhao, Qiangqiang Li, Jianfeng Zhu

**Affiliations:** 1 Department of Eye Disease Prevention, Shanghai Eye Disease Prevention & Treatment Center, Shanghai, China; 2 Department of Ophthalmology, Shanghai First Peoples’ Hospital, affiliated Shanghai Jiaotong University, Shanghai, China; 3 Department of School Health, Baoshan Center for Disease Prevention and Control, Shanghai, China; Medical College of Soochow University, China

## Abstract

**Purpose:**

To evaluate the association between the AL/CR ratio and refractive state and explore the effectiveness of this ratio in the assessment of myopia, especially when combined with uncorrected visual acuity in schoolchildren among whom myopia is common.

**Methods:**

Cross sectional study. 4686 children from 6 primary schools, aged from 6 to 12 years were selected using the clustered-stratified random sampling method. Uncorrected visual acuity (UCVA), axial length (AL), corneal radius of curvature (CR), and cycloplegic refraction were tested. Refraction was measured as the spherical equivalent (SE).

**Results:**

3922 children were included in the analysis. The mean AL/CR ratio was 2.973±0.002, increased with age, and different in gender. The coefficients of correlations of the SE with the AL/CR ratio, AL, and CR were -0.811, -0.657, and 0.095, respectively. Linear regression showed a 10.72 D shift towards myopia with every 1 unit increase in the AL/CR ratio (P<0.001, r^2^ = 66.4%). The estimated SE values obtained by substituting the AL/CR ratio and gender back to the regression model that were within a difference of ±0.50 D in ATE/LER (allowable total error and limits for erroneous results) zones compared to the actual measured values was 51%. The area under the ROC curve of the AL/CR ratio, AL, and UCVA for myopia detection were 0.910, 0.822, and 0.889, respectively, and the differences between each pair were statistically significant (P<0.01). At a specificity of 90%, the sensitivities were 72.98%, 50.50%, 71.99%, and 82.96%, respectively, for the AL/CR ratio, AL, UCVA, and the combination of the AL/CR ratio and UCVA.

**Conclusions:**

The AL/CR ratio was found to explain the total variance in SE better than AL alone. The effectiveness of the AL/CR ratio was statistically significantly better than UCVA for detecting myopia in children, and combining the two produced increased sensitivity without significantly decreasing specificity.

## Introduction

Myopia is a public health problem in China and other countries in East Asia [1]. During the past several decades, the prevalence of childhood myopia has increased rapidly, the age of onset of myopia has decreased, and the severity of the myopia has increased [2–5]. In urban areas in these countries, 80–90% of children completing high school are now myopic, and 10–20% can have high myopia [3,6,7]. The most common complication of high myopia is myopic retinopathy, which is a major cause of irreversible vision loss and blindness. For these reasons, there is an extreme need to control the onset and progression of childhood myopia.

Myopia is one type of refractive error. The refractive status of human eyes is a complex variable, determined by the balance of the optical power of the cornea and the lens, and the axial length of the eye [8–11]. The essence of myopia is that the axial length grows beyond the combined optical power of the cornea and the lens. Most children are born hyperopic [12].During the first 1 to 2 years after birth, there is an active process shaping the distribution of refraction, known as emmetropization [13].After that period, the cornea is relatively stable throughout development, while axial length (AL) increases and lens power decreases. And AL is one of the key variables determining the refractive status of the eye. The correlation coefficients between AL and spherical equivalent (SE) in schoolchildren were reported in the range of 0.44–0.68[14–16].It is also widely accepted that the age-related myopic shift in schoolchildren is mainly attributable to excessive axial elongation [14–19]. AL grows beyond the length at which emmetropia occurs, and that leads to myopia. Prior to emmetropia, short axial length tends to keep hyperopia.The epidemic of myopia in China may be mostly based on a failure to keep axial length within normal limits. Control of the axial elongation of the eye during development is thus crucial to achieving non-myopia.

However, in reality, some relatively short eyes can be myopic and some relatively long eyes can be hyperopic. This turns out to be because much of the compensatory adjustment of the optical components of the eye involves interactions between axial length and corneal curvature radius (CR) during the first two years of life. By the ages of 3–5 years, eyes with mildly hyperopic refraction have compensated by increasing the AL to match the CR. Since the cornea is stable, as the AL continues to increase, the eye passes through emmetropia to become myopic.The correlation between AL and CR is strong and positive [17, 18], reaches a peak at emmetropia, and is lower for hyperopes and myopes. Grosvenor was one of the first researchers to demonstrate an association between the AL/CR ratio and refractive state [20]. The AL/CR ratio was then found to be more closely related to refraction than AL alone [14–18, 20–24].Once AL matches CR, an emmetropic refraction is produced, and as it grows further, refractions become myopic as the AL/CR ratio starts to exceed about 3.0 [23,24]. AL/CR ratios can be a useful marker of the onset and the progression of myopia.

Identifying children with myopia as early as possible is important so that myopic progression can be identified. Cycloplegic refraction is the gold standard for measuring refraction. It provides the most direct means of tracking myopia [25–27], but repeated mass monitoring using cycloplegic agents is difficult to implement and creates problems with compliance. The most commonly used approach is to monitor uncorrected visual acuity (UCVA). This is because most of the reductions in visual acuity in children are due to the development of myopia [28]. However, Children with mild-moderate hyperopia and mild astigmatism can often achieve good VA, which UCVA testing could not detect. And children with severe hyperopia and astigmatism also cause reduced VA. UCVA testing alone could not distinguish them from myopia[29, 30].Alternative non-invasive approaches include measurement of non-cycloplegic refraction(NCR), AL and the AL/CR ratio. Because of the high prevalence of myopia in schoolchildren, attempts have been made to use AL and AL/CR ratios in some parts of China in recent years[31–33].

In 2011 the Ministry of Health and Education of Shanghai, began to promote the Children Eye Care Program to establish an archive of childhood refractive development data. UCVA, visual acuity with spectacles (SVA) for those children wearing them, NCR, AL, and CR were tested in schools by public health physicians and optometric physicians. Notices of referral were issued to children who were suspected of suffering from refractive errors and insufficient correction, other eye diseases, and high risk factors(less hyperopic refraction at baseline, less outdoor times, more near works and parental myopia[27]) of myopia even if their vision was still normal. In the hospital, children with normal vision but with high risk factors for myopia, underwent cycloplegic refraction, and two preliminary prognoses were made: refractive developmental normal or refractive developmental deviation according to the cycloplegic refraction. The main purpose of this prognosis is to predict myopia and guide children’s guardians to implement prevention and control as soon as possible.

However, only a few children with normal vision followed referral advice and visited the hospital for cycloplegic treatment (around 20%). Assuming that the efficiency of the AL/CR ratio for the detection of myopia in children is acceptable, IOL-master will be considered a non-invasive basic public health service tool in school-based screening, and it will probably improve the compliance of children and their parents. Then the growth trajectory of individual refraction could be evaluated through regular detection of AL and CR instead of cycloplegic refraction. As far as we know, there have been no studies on whether the AL/CR ratio and the combination of the AL/CR ratio and UCVA are suitable for the detection of myopia in schoolchildren.

The current study focuses on the AL/CR ratio. Its primary aims are as follows: (1) Description of the AL/CR ratio and other biometric parameters of 6- to-12-year-old schoolchildren in an eastern metropolis in China. (2) Evaluation of the association between the AL/CR ratio and SE as compared to AL. (3) Exploration of the efficiency of the AL/CR ratio for detection of myopia, especially when combined with UCVA in schoolchildren with a high prevalence of myopia.

## Methods

### Ethics statement

This study was approved by the Ethics Committee of Shanghai Eye Disease Prevention and Treatment Center, and the research was conducted in accordance with the Declaration of Helsinki. The nature and possible consequences of the study were explained at each school. After the school principal agreed to participate, the details regarding the examination were explained to the parents and guardians of all children at a meeting prior to the examination, and written informed consent was obtained from each. The children provided verbal consent on the day of the examination. Examination after cycloplegia was only performed in children whose parents and guardians had agreed to their participation in all examination items. Children whose parents and guardians had agreed to participation in all examination items except for cycloplegic refraction were given examination without cycloplegia.

### Subjects

The Baoshan Eye Study is a school-based survey of eye health in a large sample of year 1 to year 5 children (6-to-12-year-olds) attending primary schools across the urban-rural fringe area of Shanghai. It is a preliminary study of a 3-year public health program designed to establish childhood refractive development archives in Shanghai. The program covers about one million children, including preschoolers as young as 3 and primary and secondary school students all over Shanghai. Its main objectives involve myopia prevention and control. According to the data provided by the education sector of Baoshan district in 2010, there were 58,000 schoolchildren aged 6 to 12 graded from 1 to 5 in 85 primary schools, including 16 private schools and 69 public schools. With a stratified-clustered sampling method, 6 primary schools (1 private school and 5 public schools) including 4686 students graded from 1 to 5 were randomly selected. This met the sample number criteria for total number of samples, providing a representative sample of Baoshan primary schools. The simple random sampling formula n = Z^2^(P) (1–P)/d^2^ was used to estimate the number of samples at 4463 by taking factors of clustering effects associated with the sampling design and stratification into consideration.

### Examination

The investigation was conducted in schools from May 2010 to April 2011 by one team with five optometrists, two public health physicians, and one ophthalmologist. An experienced public health physician specializing in the prevention of children’s eye diseases from Shanghai Eye Disease Prevention and Treatment Center was made the project coordinator and ran the whole investigation.

The examination process began with testing visual acuity at 5 m using a standard logarithmic tumbling E chart (wh01; Wehen, Guangzhou, China). Acuity was tested with and without refractive correction for those wearing spectacles. Cycloplegic autorefraction was performed with a Desktop Autorefractor ((Model No.: KR-8800; Topcon Corporation, Tokyo, Japan) with a measurement range of -20 to +20 diopter (D). This instrument provided a median value of the 3 reliable readings in each eye. These were used for analysis. Each child was reexamined if the differences between the any 2 results of the 3 results obtained were greater than 0.50 D. Cycloplegia was induced in each eye by the instillation of five drops of tropicamide 0.5% (Wuxi Shanhe Group, Jiangsu, China) 5 min apart [34, 35]. Autorefraction was performed at least 30 min after the last drop. Extra tropicamide 0.5% (1 or 2 drops) was also used in some children to obtain adequate mydriasis (a minimum pupil diameter of 6 mm or disappearance of pupillary light response). Measurements of ocular biometric parameters (axial length, keratometry) were performed with an ocular biometry system (IOLMaster; version 5.02, Carl Zeiss Meditec, Oberkochen, Germany). The subjects were required to blink prior to measurement to make sure the tear film covered the whole cornea. The measurements of AL were considered valid if individual measurements varied by no more than 0.02 mm.

Any children presenting visual acuity lower than 20/25 in either eye were given subjective optometry to obtain the best corrected visual acuity. All cases in which results could not be obtained and in which there were large differences between examination and reexamination are recorded in the remarks. Anterior segments and fundus were examined with a slit-lamp and ophthalmoscope, and abnormalities in eyelids, conjunctiva, cornea, lens, vitreous fluid, and fundus were recorded. Strabismus examination was performed using cover testing.

### Quality control

Before the start of the investigation, every member of the research team was given the same training regarding the aims, methods, and processes of the investigation. After training, at the site for another project organized by Shanghai Eye Disease Prevention and Treatment Center (include all the examination items in this investigation and the equipment and methods used were the same), the consistency of different examiners was tested in twenty 6-to-12-year-old primary schoolchildren. The average difference of the SE for right eye in autorefraction was 0.04 D (SD = 0.12 D) and for left eye was 0.01 D (SD = 0.12 D). The difference was not statistically significant according to paired T-test (for right eye, t = -1.677, *P* = 0.110; for left eye, t = -0.142, *P* = 0.888). The average intraclass correlation coefficients for AL and CR measurement were 0.995 and 0.989, respectively. The same equipment was used throughout the investigation. All equipment was checked and calibrated daily. During the investigation, the project coordinator supervised every step to avoid deviation from the standard during operation. During the inspection phase, the coordinator reviewed the record form. Any missing entries were eventually addressed. Unreliable results were addressed by reexamination.

### Definitions and data analysis

A database was established using Epidata3.1 and data entry and logic verification were conducted. Then statistical analysis was performed using commercial software (SAS software ver.9.1.3; SAS, Cary, NC, U.S.).

SE = spherical degree + 0.5 × cylinder degree. The axial length-corneal radius (AL/CR ratio) was defined as the axial length divided by the mean corneal radius of curvature.Myopia was defined as the SE≤-0.50 D,emmetropia as -0.50 D<SE<+0.50 D, and hyperopia as SE≥+0.50 D [14]. The definition of amblyopia is taken from the Refractive Error Study in Children (RESC) surveys [36]. It was considered with best-corrected visual acuity ≤ 20/40 and no apparent organic lesion if one or more of the following criteria were met: (1) esotropia, exotropia, or vertical tropia at 4-m fixation or exotropia or vertical tropia at 0.5 m,(2) anisometropia of 2.00 SE D or more, or (3) bilateral ametropia of at least +6.00 SE D.

The quantitative indicators are presented here as mean values ± standard deviation (mean±SD) and qualitative indicators are described in relative terms. Comparisons of quantitative indicators between different groups were checked with a χ^2^ test and comparisons of the mean values of all the indicators between different groups were checked with independent sample t-testing or analysis of variance. A normality test (Kolmogorov-Smirnov test) was performed prior to analysis of the correlations between SE, AL, the AL/CR ratio, and CR. This also provided the values of skewness and kurtosis. Skewness is a measure of symmetry. A zero skewness value indicates that the tails on both sides of the mean balance out, and a positive skew indicates that the tail on the right side is longer or fatter than the one on the left side. Kurtosis is a measure of whether the data are peaked or flat relative to a normal distribution. Data sets with high kurtosis tend to have distinct peaks near the mean, decline somewhat rapidly, and have heavy tails. Data sets with low kurtosis values tend to have flat tops near the mean rather than sharp peaks.

When the variables met or approximately met normal distribution, simple linear pearson correlation analysis was performed, otherwise performed with rank spearman correlation analysis. The efficiency of AL and the AL/CR ratio in predicting the SE and the influence of age on the prediction were analyzed by fitting multiple linear regression models. The consistency between the SE values obtained by predicting the SE regression curve with the AL/CR ratio and the actual SE values were checked with ATE/LER (allowable total error and limits for erroneous results) zones [37]. A plot scatter map with the groups of data was made. Then the equal line was drawn such that it would meet y = x. After drawing a line parallel to y = x±m (m represents clinically acceptable error), the relative number of points that fell within the ATE zones was calculated. Higher percentages indicate better consistency between the two methods. The diagnostic test for optimal cutoff point of myopia detection was performed using ROC curves (receiver operating characteristic). The point on the curve nearest the vertex located in the upper-left corner was found and then the sensitivity, specificity, Youden index, and other values were calculated. All confidence intervals (CI) are 95%. *P* values were considered significant at the 0.05 level.

## Results

### Sample population characteristics

Among the 4686 sampled and registered children who planned to undergo examinations, 4118(87.9%) agreed to undergo all the examinations including cycloplegic refraction, and 568 (12.1%) children agreed to undergo some examinations but not cycloplegic refraction. On the day the examinations were performed, the actual number of children who participated in the examinations was 4594 (98.0%),and children who completed cycloplegic refraction was 3975 (84.8%). Because the SE and AL of the right eye are closely related to those of the left eye (Pearson coefficient: SE = 0.912, AL = 0.953, *P*<0.001), only data from the right eye were included in the analysis.

The difference in the mean AL, CR, and AL/CR values of the right eye and the gender distribution of the children who completed cycloplegic refraction were not statistically significantly different from those who had not undergo cycloplegic refraction (619) (AL: t = -1.481, *P* = 0.139; CR: t = -1.370, *P* = 0.171; AL/CR: t = -0.402, *P* = 0.688; gender: χ^2^ = 0.321, *P* = 0.571), and the difference in the mean of age was statistically significant (mean±SD with and without cycloplegic refraction were 8.89±1.63 and 9.10±1.62, respectively, t = -2.928, *P* = 0.003). With exclusion of the cases ofmissing, amblyopia, obvious strabismus, ocular atrophy, cataracts, data from 3922 children were included in the analysis. Among these, 2106 (53.7%) were boys and 1816 (46.3%) were girls,aged 9.11±1.64 and 9.07±1.60, respectively, and the difference was not statistically significant (t = 0.639, *P* = 0.488). The age and gender of the children actually included in the investigation were not statistically significantly different from those of the subjects who had planned to be included in the investigation but did not undergo testing (Table 1). This indicated that the sample included in the analysis was representative.

**Table 1:** Enumerated and examined population by age and gender.

Age (yrs)	Enumerated (%)	Examined		informed consent for cycloplegia		Cycloplegic autorefraction	
		No. (%)	Percent	No. (%)	Percent	No. (%)	Percent
6^a^	111 (2.4)	108 (2.4)	97.3	88 (2.1)	79.3	85 (2.1)	76.6
7	865 (18.5)	838 (18.2)	96.9	737 (17.8)	85.2	702 (17.7)	81.2
8	954 (20.4)	935 (20.4)	98.0	841 (20.3)	88.2	814 (20.5)	85.3
9	852 (18.2)	842 (18.3)	98.8	760 (18.3)	89.2	737 (18.5)	86.5
10	854 (18.2)	838 (18.2)	98.1	762 (18.4)	89.2	735 (18.5)	86.1
11	787 (16.8)	772 (16.8)	98.1	688 (16.6)	87.4	666 (16.8)	84.6
≥12^b^	263 (5.6)	261 (5.7)	99.2	242 (5.8)	92.0	236 (5.9)	89.7
Boys	2532 (54.0)	2468 (53.7)	97.5	2236 (54.3)	88.9	2142 (53.9)	84.6
Girls	2154 (46.0)	2126 (46.3)	98.7	1882 (45.7)	87.8	1833 (46.1)	85.1
Total	4686 (100.0)	4594 (100.0)	98.0	4118(100.0)	87.9	3975 (100.0)	84.8

^a^ This age group with enumerated cycloplegic refraction data includes one 5-year -old child.

^b^ This age group with cycloplegic refraction data includes fifty-five 13-year-old children and eleven 14-year-old children.

The age and sex distribution of those examined and those who provided informed consent for cycloplegia and received cycloplegic autorefraction were not significantly different from those of the rest of the population (chi-square goodness of fit, *P*>0.05).

### Prevalence of myopia

The prevalence of myopia in the total was 31.1%, higher among older participants than among younger participants, was 11.1% in 6-to-7-year-olds,19.4% in 8-year-olds,28.0% in 9-year-olds,44.0% in 10-year-olds, 51.2% in 11-to-12-year-olds. Among the total, 85.0% had mild myopia (-3.00 D<SE≤-0.50 D). The prevalence was also higher in girls than in boys (30.1% vs. 26.4%). The mean refractive was more myopic in girls than in boys (-0.10 D vs. 0.00 D).

### Distribution of AL, CR, AL/CR ratio, and SE

Table 2 shows in the sample of 6- to-12-year-old children, kurtosis was greatest for the distribution of the SE (kurtosis 4.97), more normal distributions for all other ocular biometric parameters (kurtosis -0.04–0.68). Distributions of AL and CR appeared normal in specific age groups but overall. Skew for the SE was -0.84, and ranged from 0.11 to 0.50 for the other ocular biometric parameters. Values of both AL and the AL/CR ratio increased with age,by 0.18–0.27 mm and 0.02–0.04 every year, respectively. SE values decreased as age increased, by 0.24–0.43 D every year, and the difference was statistically significant (*P*<0.001). The mean SE values in the 11-year-old children (-0.68 D) had exceeded the diagnostic criteria of myopia(≤-0.50 D). Only the mean value of CR remained stable regardless of increases in age (*P* = 0.720). The differences between the boys and girls in the mean values of AL, CR, AL/CR ratio, and SE were statistically significant (*P*<0.001). The mean values of AL, CR and the AL/CR ratio in boys were higher than in girls. The mean SE in boys was more hyperopic, 0.10 D higher than in the girls.

**Table 2:** Measures of spread for refraction (SE) and ocular biometric parameters in the right eyes of children by age and gender.

	Minimum	25%	Median	75%	Maximum	Mean∮	SE	SD	Skewness	Kurtosis	K-S#	
											Z	*P*
**AL**												
Age(yrs)												
6–7	19.75	22.42	22.88	23.33	25.73	22.88A	0.03	0.74	-0.14	0.79	0.85	0.47
8	20.54	22.62	23.10	23.64	25.95	23.11B	0.03	0.80	-0.02	0.28	0.92	0.37
9	20.56	22.72	23.25	23.83	26.96	23.29C	0.03	0.87	0.27	0.54	1.25	0.09
10	20.41	22.97	23.53	24.11	26.67	23.56D	0.03	0.90	0.10	0.33	0.66	0.77
≥11	20.79	23.16	23.77	24.36	27.40	23.79E	0.03	0.97	0.29	0.69	1.08	0.19
Boys	19.91	22.99	23.49	24.08	27.40	23.56*	0.02	0.88	0.35	1.02	1.57	0.01
Girls	19.75	22.49	23.03	23.64	27.29	23.08*	0.02	0.90	0.34	0.56	1.71	0.01
Total	19.75	22.73	23.28	23.91	27.40	23.34	0.01	0.92	0.30	0.68	2.18	<0.01
**CR**												
Age (yrs)												
6–7	7.07	7.68	7.85	8.04	8.71	7.86A	0.01	0.26	0.01	-0.27	1.03	0.24
8	7.07	7.68	7.87	8.04	8.65	7.86A	0.01	0.26	0.09	0.02	1.32	0.06
9	7.11	7.65	7.85	8.01	8.77	7.84A	0.01	0.25	0.13	-0.13	0.95	0.33
10	7.14	7.67	7.83	8.01	8.82	7.84A	0.01	0.26	0.21	0.11	0.95	0.33
≥11	7.20	7.69	7.85	8.01	8.79	7.86A	0.01	0.26	0.14	0.07	1.06	0.21
Boys	7.07	7.76	7.89	8.08	8.82	7.91*	0.01	0.25	0.06	0.11	1.55	0.02
Girls	7.07	7.61	7.78	7.94	8.65	7.78*	0.01	0.25	0.22	-0.03	1.42	0.03
Total	7.07	7.67	7.85	8.04	8.82	7.85	0.00	0.26	0.11	-0.04	1.97	<0.01
**AL/CR ratio**												
Age (yrs)												
6–7	2.60	2.86	2.91	2.96	3.21	2.913A	0.003	0.08	0.24	1.32	1.39	0.04
8	2.62	2.89	2.94	2.99	3.27	2.940B	0.003	0.09	-0.05	1.33	1.49	0.02
9	2.64	2.91	2.96	3.02	3.41	2.971C	0.004	0.10	0.58	1.52	1.70	0.01
10	2.61	2.94	2.99	3.07	3.39	3.006D	0.004	0.11	0.35	0.93	1.75	<0.01
≥11	2.58	2.96	3.02	3.09	3.44	3.030E	0.004	0.11	0.23	1.25	1.43	0.03
Boys	2.58	2.91	2.97	3.03	3.41	2.979*	0.002	0.10	0.53	1.49	3.11	<0.01
Girls	2.59	2.90	2.95	3.03	3.44	2.966*	0.002	0.11	0.50	0.95	2.29	<0.01
Total	2.58	2.90	2.96	3.03	3.44	2.973	0.002	0.11	0.50	1.20	3.59	<0.01
**SE**												
Age (yrs)												
6–7	-5.50	0.13	0.63	1.00	7.88	0.58A	0.03	0.97	-0.07	9.58	3.45	<0.01
8	-5.00	0.00	0.50	0.88	7.00	0.34B	0.04	1.09	0.19	6.36	3.70	<0.01
9	-10.25	-0.50	0.25	0.75	6.25	0.01C	0.05	1.32	-1.08	7.91	4.13	<0.01
10	-7.75	-1.13	-0.13	0.50	6.88	-0.42D	0.05	1.44	-0.58	3.20	3.52	<0.01
≥11	-6.75	-1.28	-0.31	0.25	7.38	-0.68E	0.06	1.67	-0.63	3.34	3.65	<0.01
Boys	-10.25	-0.50	0.25	0.75	7.38	0.00*	0.03	1.36	-0.79	6.47	6.67	<0.01
Girls	-7.75	-0.75	0.13	0.75	7.88	-0.10*	0.03	1.47	-0.86	3.60	5.79	<0.01
Total	-10.25	-0.63	0.25	0.75	7.88	-0.05	0.02	1.41	-0.84	4.97	8.81	<0.01

#K-S, Kolmogorov-Smirnov test for normality

∮ Means with different letters (A,B,C,D, and E) were significantly different (*P*<0.05, ANOVA, LSD)

* *P*<0.05, independent Samples T Test.

### Relationship between SE, AL, CR and AL/CR ratio

**Fig 1:**
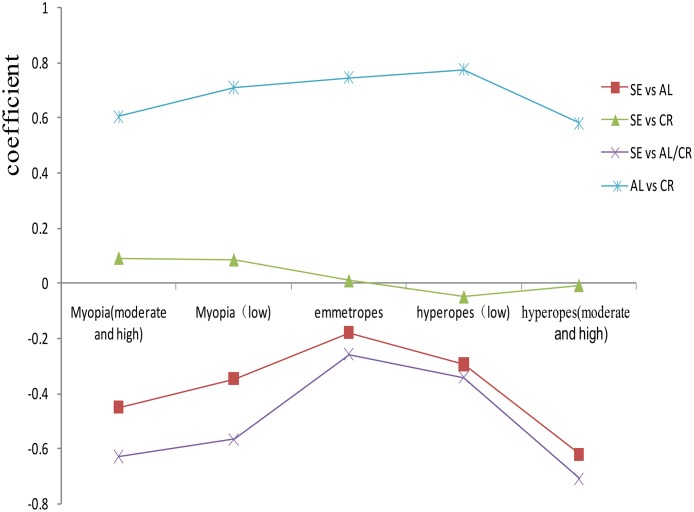
Correlation by refractive state. Myopia (moderate and high): SE≤-3.00D; Myopia (low): -3.00 D<SE≤-0.50 D; emmetropes: -0.50 D<SE<+0.50 D; hyperopes (low): +0.50 D≤SE<+2.00 D; hyperopes (moderate and high): SE≥+2.00 D.

**Fig 2:**
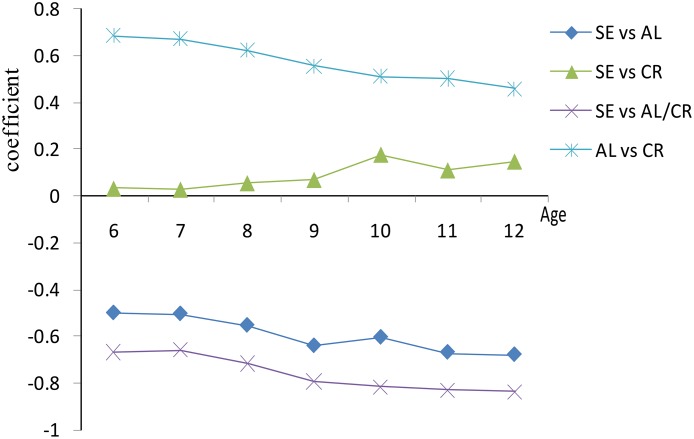
Correlation by age and gender.

As indicated in Table 3, the mean AL value of myopic children was 0.80 mm higher than in emmetropic children and the mean AL value of hyperopic children was 0.44 mm lower than in emmetropic children. The mean AL/CR ratio value of myopic children was 0.08 higher than in emmetropic children and the mean AL/CR ratio of hyperopic children was 0.06 lower than in emmetropic children. The mean CR value of myopic children was 0.03～0.04 lower than that of emmetropic and hyperopic children. The coefficients of pearson correlation between SE and AL and between SE and the AL/CR ratio were -0.657 and -0.811 (*P*<0.001), respectively. The correlation between SE and CR was low, but significant, giving a coefficient of 0.095 (*P*<0.001). The coefficient between SE and age was -0.321 (*P*<0.001). In different refractive status, the correlation coefficients between SE and AL, AL/CR ratio were lower in emmetropes than in hyperopes and myopes, and the correlation coefficient between AL and CR was higher in emmetropes than in hyperopes and myopes (Fig. 1). However, \ the correlation coefficients between SE and AL, the AL/CR ratio increased with age, and the correlation between AL and CR decreased with age (Fig. 2). The differences among correlation coefficients between SE, AL, the AL/CR ratio, and CR and between AL and CR in different gender groups were not statistically significant (*P*>0.05).

**Table 3:** Mean values of AL, CR and AL/CR ratio according to refractive status.

		N	AL			CR				AL/CR ratio		
			Minimum	Maximum	Mean±SD*	Minimum	Maximum	Mean±SD*		Minimum	Maximum	Mean±SD*
Myopia	Total	1103	21.00	27.40	24.09±0.87	7.09	8.82	7.82±0.25		2.74	3.44	3.08±0.10
	≤-3.00D	166	23.06	27.40	24.99±0.85A	7.20	8.44	7.78±0.24	A	2.81	3.44	3.21±0.09A
	>-3.00D–≤-0.50D	937	21.00	27.33	23.93±0.77B	7.09	8.82	7.82±0.25	B	2.74	3.31	3.06±0.08B
Emmetropia	>-0.50D–<+0.50D	1249	21.00	25.75	23.29±0.70C	7.14	8.77	7.86±0.25	C	2.68	3.37	2.96±0.07C
Hyperopia	Total	1570	19.75	25.28	22.85±0.75	7.07	8.65	7.87±0.27		2.58	3.16	2.90±0.07
	≥+0.50D–<+2.00D	1464	20.55	25.28	22.91±0.71D	7.07	8.65	7.87±0.27	C	2.63	3.16	2.91±0.06D
	≥+2.00D	106	19.75	23.42	21.92±0.78E	7.28	8.60	7.90±0.25	C	2.58	2.98	2.78±0.09E

* Means with different letters (A, B, C, D, and E) were significantly different (*P*<0.05, ANOVA, LSD)

(ANOVA, AL:F = 593.899, *P*<0.001; CR:F = 9.339, *P*<0.001; AL/CR ratio: F = 129.527, *P*<0.001; SE:F = 1366.419, *P*<0.001)

### Regression analysis conducted to estimate the relationships among SE, AL and the AL/CR ratio

**Fig 3:**
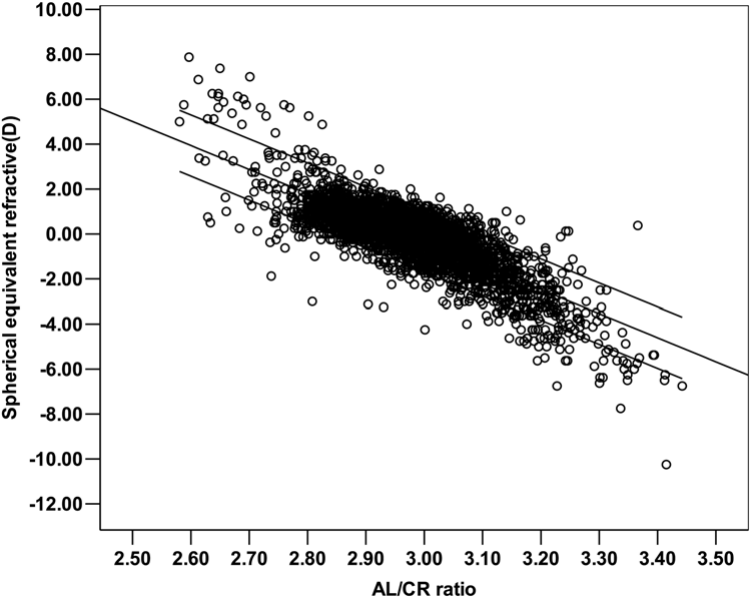
Regression line of the AL/CR ratio and SE with the 95% confidence interval. SE = 31.672–10.656AL/CR ratio (r = -0.811, r2 = 65.7%, *P*<0.001).

**Fig 4:**
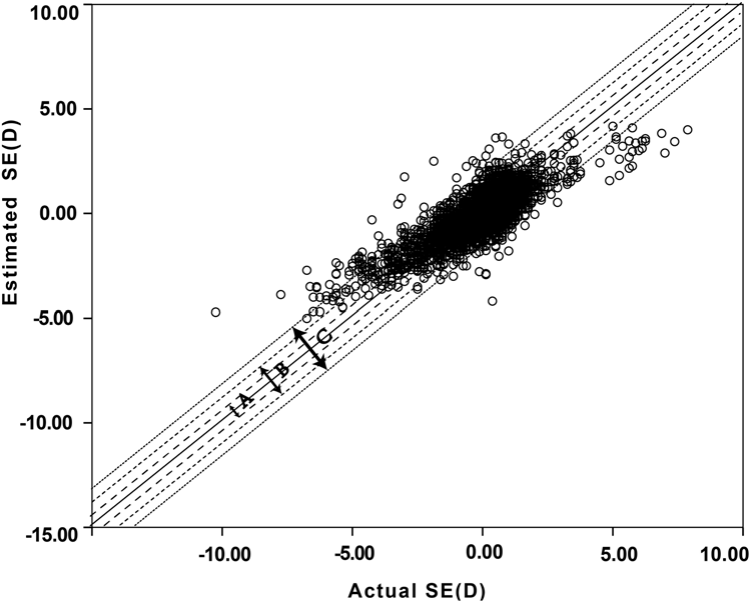
ATE/LER zones of the actual measured SE values and estimated SE values. (Estimated using SE = 32.208–10.724AL/CR-0.234Gender). A: 51.0% (+-0.50D), B: 82.2% (+-1.00D), C: 93.5% (+-1.50D).

Table 4 indicated that AL, age, and gender could explain 48.3% of the total variance in SE (F = 1219.80, *P*<0.001). Age and gender accounted for 5.2% of the variation.SE and AL indicated that SE would change by 1.01 D as the AL changed by 1.0 mm. When multiple linear regression analysis was conducted for SE, AL/CR ratio, age, and gender, the AL/CR ratio could explain 65.7% of the total variance in SE (F = 2579.09, *P*<0.001). Varying the AL/CR ratio by 0.1 caused the corresponding SE to change by 1.07 D (Fig. 3).The estimated SE values were obtained by substituting the AL/CR ratio and gender back to the regression model (SE = 32.208–10.724AL/CR-0.234Gender). As shown in Fig. 4, 51% of estimated SE values differed from the measured values by no more than ±0.50 D. These results are better than those produced by AL but not strong enough to show that the two values are in close agreement.

**Table 4:** Linear regression analysis of SE and AL, AL/CR.

Item	B	Standardized coefficients	95.0% confidence interval for B	t	*P*
SE and AL, age, gender (F = 1219.80, *P*<0.001, r = 0.695, r2 = 0.483)					
Constant	25.84		25.395–26.285	58.032	<0.001
Age	-0.077	-0.089	-0.088–-0.067	-7.226	<0.001
Gender#	-0.599	-0.212	-0.633–-0.565	-17.737	<0.001
AL	-1.042	-0.681	-1.061–-1.022	-53.543	<0.001
SE and AL/CR, age, gender (F = 2579.09, *P*<0.001, r = 0.815, r2 = 0.664)					
Constant	32.208		31.833–32.584	85.774	<0.001
Age	-0.003	-0.004	-0.012–0.005	-0.373	0.709
AL/CR	-10.724	-0.814	-10.857–-10.591	-80.736	<0.001
Gender#	-0.234	-0.083	-0.260–-0.208	-0.898	<0.001

# categorical variable, boys as the reference level

### Diagnostic tests for detecting myopia with the AL/CR ratio, AL, UCVA, and combined use of UCVA and the AL/CR ratio

**Fig 5:**
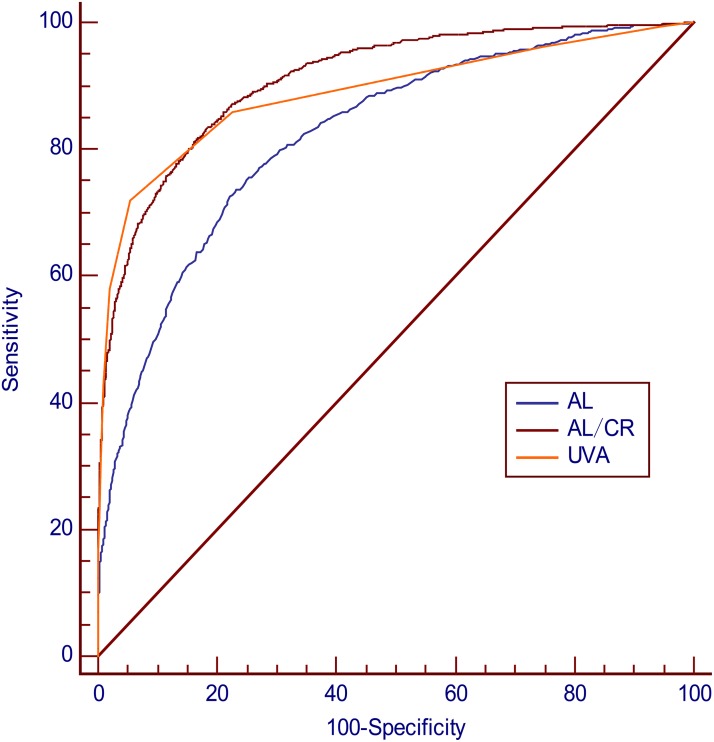
Comparison of receiver operating characteristic (ROC) curves among AL/CR, AL, and UCVA for the detection of myopia in schoolchildren. AUC (95%CI): AL/CR, 0.910 (0.901–0.919); AL, 0.822 (0.810–0.834); UCVA, 0.889 (0.879–0.899). AUC, area under the ROC curve; AL, axial length; AL/CR, axial length/corneal radius ratio; UCVA, uncorrected visual acuity.

Cycloplegic SE ≦ -0.50 D is the gold standard for diagnosis of myopia. ROC curves were plotted using the AL/CR ratio, AL, and UCVA as indicators of myopia, and the areas under the ROC curve were 0.910 (95%CI 0.901–0.919),0.822 (95%CI 0.810–0.834), and 0.889(95%CI 0.879–0.899), respectively (Fig. 5). The differences between areas in each pairwise were statistically significant (Z test, *P*<0.01). For the AL/CR ratio, the optimal cutoff point for myopia detection was >2.99, with a sensitivity and specificity of 83.05% and 81.91%, respectively. For AL, the optimal cutoff point was >23.59 mm, with a sensitivity and specificity of 72.62% and 77.76%, respectively. For UCVA, the optimal cutoff point was ≤20/32 with a sensitivity and specificity of 72.19% and 94.47%, respectively (Table 5). When UCVA was combined with the AL/CR ratio, optimal cutoff point the optimal cutoff point was UCVA ≤20/25 and the AL/CR ratio >2.95, with sensitivity and specificity of 82.96% and 90.56%, respectively. At a specificity of 90%, the sensitivities were 71.99%, 72.98%, and 82.96%, respectively, for UCVA, the AL/CR ratio, and the combination test (Table 6) [38,39].

**Table 5:** Sensitivity, specificity, PPV, NPV, 95% CI, and Youden Index of UCVA, AL, and AL/CR of screening of schoolchildren for myopia.

Criterion	Sensitivity (95% CI)	Specificity (95% CI)	PPV (95% CI)	NPV (95% CI)	Youden index
AL/CR					
>2.94	96.01 (94.7–97.1)	54.74 (52.9–56.6)	45.4 (43.3–47.4)	97.2 (96.3–98.0)	0.5075
>2.96	92.66 (91.0–94.1)	66.19 (64.4–67.9)	51.7 (49.5–54.0)	95.8 (94.9–96.7)	0.5885
>2.98	88.03 (86.0–89.9)	75.56 (73.9–77.1)	58.5 (56.1–60.9)	94.2 (93.1–95.1)	0.6359
>3.00	81.05 (78.6–83.3)	83.82 (82.4–85.2)	66.2 (63.6–68.7)	91.9 (90.8–92.9)	0.6487
>3.02	72.98 (70.3–75.6)	90.10 (88.9–91.2)	74.3 (71.5–76.8)	89.5 (88.3–90.6)	0.6308
>3.04	64.82 (61.9–67.6)	94.29 (93.4–95.1)	81.6 (78.9–84.1)	87.3 (86.0–88.4)	0.5911
>3.06	56.48 (53.5–59.4)	96.81 (96.1–97.4)	87.4 (84.7–89.7)	85.0 (83.8–86.3)	0.5329
AL					
>23.00	90.75 (88.9–92.4)	47.25 (45.4–49.1)	40.2 (38.3–42.2)	92.9 (91.4–94.2)	0.3800
>23.20	85.95 (83.8–87.9)	58.96 (57.1–60.8)	45.0 (42.9–47.2)	91.5 (90.1–92.7)	0.4491
>23.40	79.33 (76.8–81.7)	69.78 (68.0–71.5)	50.7 (48.3–53.0)	89.6 (88.3–90.9)	0.4911
>23.60	71.80 (69.0–74.4)	78.15 (76.6–79.7)	56.2 (53.6–58.9)	87.6 (86.3–88.9)	0.4995
>23.80	61.92 (59.0–64.8)	84.57 (83.2–85.9)	61.1 (58.2–64.0)	85.0 (83.6–86.3)	0.4649
>24.00	50.50 (47.5–53.5)	90.03 (88.9–91.1)	66.5 (63.2–69.7)	82.3 (80.9–83.6)	0.4053
>24.20	42.07 (39.1–45.0)	93.37 (92.4–94.3)	71.3 (67.6–74.7)	80.5 (79.1–81.8)	0.3544
UCVA					
≤20/63	30.55 (27.8–33.4)	99.47 (99.1–99.7)	95.7 (93.1–97.6)	78.5 (77.2–79.9)	0.3002
≤20/50	42.61 (39.7–45.6)	99.04 (98.6–99.4)	94.6 (92.2–96.4)	81.5 (80.2–82.8)	0.4165
≤20/40	58.11 (55.1–61.0)	97.87 (97.3–98.4)	91.4 (89.1–93.4)	85.7 (84.4–86.8)	0.5598
≤20/32	71.99 (69.2–74.6)	94.43 (93.5–95.2)	83.5 (81.0–85.8)	89.6 (88.4–90.7)	0.6642
≤20/25	86.04 (83.9–88.0)	77.37 (75.8–78.9)	59.8 (57.3–62.2)	93.4 (92.3–94.4)	0.6341
≤20/20	96.28 (95.0–97.3)	25.12 (23.5–26.8)	33.5 (31.8–35.1)	94.5 (92.6–96.0)	0.2140
≤20/16	99.55 (98.9–99.9)	5.21 (4.4–6.1)	29.1 (27.7–30.6)	96.7 (92.5–98.9)	0.0476

Area under the ROC curve, AUC (95%CI): AL/CR, 0.910 (0.901–0.919); AL, 0.822 (0.810–0.834); UCVA, 0.889 (0.879–0.899). Pairwise comparison of ROC curves: AL/CR vs. AL, z = 10.149. *P*<0.001; AL/CR vs. UCVA, z = 3.019. *P* = 0.003; AL vs. UCVA, z = 7.704. *P*<0.001.

**Table 6:** Sensitivity, specificity, and criteria for UCVA, AL/CR, and combined use of UCVA and AL/CR when specificity is set to 90% (N = 3922).

	Sensitivity (%)	Specificity (%)	Youden Index	Criteria
UCVA	71.99	94.43	0.6642	UCVA≤20/32
AL/CR	72.98	90.10	0.6308	AL/CR>3.02
Combination1*	81.69	91.42	0.7310	UCVA≤20/25 and AL/CR>2.96
Combination2*	82.96	90.56	0.7352	UCVA≤20/25 and AL/CR>2.95
Combination3*	83.95	89.29	0.7324	UCVA≤20/25 and AL/CR>2.94

*Combination means combined use of UCVA and AL/CR in serial order (children were referred if they failed both tests).

## Discussion

In this investigation, we found that the correlation coefficient of the AL/CR ratio with SE was bigger than that of AL with SE, AL/CR ratio could explain the total variance in SE better than AL alone. The AL/CR ratio could function relatively efficiently as a myopia detection indicator, and the best diagnostic cutoff value was >2.99. Its effectiveness for detecting myopia was statistically significantly better than UCVA. When the two methods were combined, even higher sensitivity was observed, with no significant drop in specificity.

The prevalence of myopia was in the “medium” level in East Asia, increased from 11.1% in 6-to-7-year-olds to 51.2% in 11-to-12-year-olds.It was higher than that in coastal eastern of China [7] and urban southern of China[40], the prevalence of myopia in 7-year-old children of which were 7.8%, 7.7%, respectively. And it was lower than that in Hongkong[41], Singapore[42], Taiwan[3], the prevalence of myopia in 7-year-old children of which were 17.0% (approximately), 29.0%,20.0%, respectively. Among myopic children, 85.0% had mild myopia, suggesting that conducting myopia detection in this population and giving early intervention would greatly contribute to the control of myopia. The prevalence of myopia was higher in girls than in boys, and the mean refractive was more myopic in girls. These were consistent with those reported in related studies [40,43].

Basic data regarding SE, AL, CR, and the AL/CR ratio of 6- to-12-year-old schoolchildren are presented in this study. This may supplement the current, limited data regarding ocular biometric parameters in East Asian individuals. As indicated in this investigation, the values of AL and CR in each age group displayed a normal distribution, while in total and in each gender displayed a skewed fashion. That may be attributed to analysis on mixed age samples by gender and total will tend to underestimate kurtosis and over-estimate skew. The values of AL/CR ratio and SE were distributed mildly skew. However, the distribution of the AL/CR ratio was regarded approximately normal as the kurtosis was also relatively low. The kurtosis of SE was relatively steeper.

Just as Twelker reported, the AL of the 6- to-12-year-old children increased with age, and the AL of the boys was 0.5 mm longer than that of the girls [44]. This manifestation was consistent with the differences in height between boys and girls [45, 46]. The AL of the 7- to-9-year-old children was lower than that reported by Saw in 7- to-9-year-old children in Singapore [42], but the mean of AL of the 6- to-7-year-old children (22.88 mm) was larger than that (22.61 mm) reported by Ojaimi in Australia [14]. This could be because the prevalence of myopia is higher in Singapore and lower in Australia than in Shanghai. CR remained stable across age groups, and higher in boys than in girls, as reported by Zadnik et al. [5, 15, 47, 48]. In this way, the AL/CR ratio increased with age and with the elongation of AL. The AL/CR ratio in the 6-year-old group of this investigation was 2.903, which gradually increased to 3.030 in the 12-year-old group. The AL/CR ratio was higher in boys than in girls. This was not consistent with that the mean SE was more myopic in girls than in the boys as well as the prevalence of myopia. It suggests there may be other biometric parameters joined into the shaping of refraction. Investigations found lens power may play an important role during refractive development [49–50]. Lens power decreases substantially up to the age of about 12 years [49], with slower decreases for most of adult life[50]. The reductions in lens power could largely neutralize the myopic shifts, which would otherwise be associated with axial elongation [51].

In this investigation, the correlation coefficient between AL and SE in 6--to-12-year-old children was -0.657. However, the AL/CR ratio showed a closer correlation with SE than AL alone, with a correlation coefficient of -0.811. This was consistent with recent previous reports [14–18, 20–22]. Grosvenor, González, and Eghosasere reported that the correlation coefficients between AL/CR ratio and SE in young adults were -0.84, -0.89, and -0.78, respectively [17, 18, 21]. In schoolchildren, Ojaimi, Ip, and Kimura found the correlation coefficients between the AL/CR ratio and SE to be -0.66, -0.61, and -0.78, respectively [14–16]. The AL/CR ratio and AL showed some similar characteristics in this investigation. The mean AL/CR ratio was higher in the higher age group and the correlation with SE increased with age. In different refractive states, the AL/CR ratio showed a low correlation with SE in emmetropic children and higher correlation in moderately hyperopic and myopic children. During the first year or two after birth, a major feature of eye development appears to be the matching of AL to CR, which appears as a marked kurtosis in the distribution of SE and the AL/CR ratio, and the distributions of AL and CR remain relatively normal. During this phase, the process narrows the distribution of SE, so the correlations are relatively low. After that, CR remains stable, but axial length continues to increase, causing increases in the AL/CR ratio, which parallel the development of myopia. This almost entirely explains why the correlations are higher for hyperopes, and increase with age and more myopic. Another concern was that the correlation between AL and CR was higher in emmetropes than in hyperopes and myopes. The correlation of AL and CR must change because CR is stable but AL increases, and more children become myopic. The correlation between AL and CR is lower when most children are hyperopic, increases as more children become emmetropic, and then falls again as more children become myopic.

Linear regression analysis of the relationship between the SE, AL/CR ratio, and age indicated that the AL/CR ratio could explain 65.7% of the total variance in SE regardless of the age. This is much higher than the 43.1% attributable to AL. However, when the AL/CR ratio was substituted back into the regression model and the SE was calculated, 51% of estimated values were found to differ from calculated values by no more than ±0.50 D. For the SE, variation of 0.50 D is considered clinically significant. For this reason, the AL/CR ratio was considered insufficiently precise for quantitative estimation of the actual refractive state at the cross-sectional level.

Grosvenor and Goss believed that the AL/CR ratio could serve as a risk factor for the development of myopia [20, 23, 24]. They considered that when the AL/CR ratio was higher than 3, the children were very likely to suffer from myopia. AL/CR ratios equal to or higher than 3 were considered indicative of a lack of compensation by other parameters and induced myopia. As demonstrated here, as an indicator of qualitative detection for myopia, the AL/CR ratio was highly precise, and the area under the ROC reached 0.910 in the total sample population. This was higher than that of AL, and better than that of UCVA (0.889). The optimal cutoff value was >2.99, which was near the cutoff value (3.0) found by Grosvenor and Goss [23, 24]. Besides, the differences between areas the under ROC curve for different age and gender groups were not statistically significant. The Youden index was within 0.60–0.67 across different age groups. This was higher than the Youden index of 0.56 reported by Goss (sensitivity 88% and specificity 68%) [23].The best diagnostic cutoff value was>2.99. This was similar to the results by Grosvenor and Goss [23, 24], and showed that the AL/CR ratio can function relatively efficiently as a myopia detection indicator.

Researchers found that the combined use of UCVA and NCR may achieve higher sensitivity than either of the two tests alone for the classification of refractive errors [52, 53].When the AL/CR ratio was combined with UCVA for detection of myopia, the Youden Index of the combination of AL/CR (0.735) and UCVA was found to be similar to the combination of NCR and UCVA (0.749) [53]. The AL/CR ratio was also found to help UCVA achieve more satisfactory sensitivity without significantly decreasing specificity. As large-scale screening settings, there was here found to be no significant difference between the IOLMaster instrument and auto-refractor with respect to their size, the difficulty of transportation, operative difficulty index, and time consumed for operation. Nurses and other public health workers are able to master it after a small amount of training. That means the main difference between the two combinations may be the cost of the instruments (approximately 100,000 RMB for refractor and 400,000 RMB for IOLMaster).

If the cost of IOLMaster is not prohibitive, we proposed that IOL-master equipment, as well as visual charts, should be brought to schools. The combination of the AL/CR ratio and UCVA could be a viable option for the detection of myopia in schoolchildren. Furthermore, AL and the AL/CR ratio have the advantage of enabling dynamic tracing and observation of the development of refraction longitudinally. These can serve as objective indicators for the prediction of myopia and the outcomes evaluated of myopia intervention projects, especially for regions in which cycloplegic refraction is difficult to implement. NCR test results can be greatly influenced by eye accommodation in children. Results may differ considerably at different times in the same day. At the cross-sectional level and in populations with high rates of myopia, it can screen for myopia very efficiently. However, as a longitudinal indicator with respect to tracing and observation, it is not stable, and its directions of change do not always reflect the truth.

## Limitations

This investigation has certain limitations. First, the mydriatic agent used in this investigation was 0.5% tropicamide, while cyclopentolate is the preferred mydriatic agent and has become the gold standard abroad. 0.5% tropicamide but not cyclopentolate is in common clinical use in China. This has caused much inconvenience in the evaluation of otherwise related ophthalmic epidemiological investigations. Although the paralytic effects of tropicamide on ciliary muscle are slightly weaker than cyclopentolate, recent studies have shown that clinically required ciliary muscle paralysis could be achieved by administration of tropicamide, and the difference between tropicamide and cyclopentolate was not statistically significant for diagnosis of myopia[34, 35]. Tropicamide especially showed advantages in shorter pupil recovery time and less severe side effects.

Second, the AL/CR ratio can explain the majority of the total variance in SE but not all of it. Recent studies have indicated that prior to the onset of myopia, because of the speed of the variations in AL and refraction, the original compensation by flattening and thinning of lens to resist the elongation of AL can disappear suddenly, which is closely related to the onset of myopia [51,54]. For this reason, there is a need to fit model combining other parameters, like lens power to completely interpret the total variance in SE.

Third, this investigation is a cross-sectional study and cannot elucidate the variations between AL and the AL/CR ratio prior to the onset of myopia. A cohort study indicated that changes in AL and SE accelerated early during the onset of myopia [32,55]. In studies conducted in 7- to-15-year-old twins, Xiang found that AL and the AL/CR ratio increased by 0.43 mm and 0.05, respectively, within the year of onset of myopia [32]. To establish reference ranges of the annual change values of AL and AL/CR in “healthy” children could help to detect children with high risk of myopia in practice. That could be determined after further investigation, and this could provide basis for the regular supervision of individuals with refraction. The children should be examined once every six months. Archives of ocular refractive development and changes in biometric parameters could also be established [56]. By dynamically tracing the changes in growth rates of AL and the AL/CR ratio, the key point in time for preventing myopia could be identified, and this could be used to determine the proper stage for myopia prevention and contribute to the control of myopia in children.

At present, methods of addressing myopia other than optical correction remain questionable. Although many studies have failed to come to a conclusive and unified final conclusion, some have recorded some useful findings. For example, outdoor activities might prevent incident myopia and slow its progression [57,58]. Although the long-term side effects of atropine and OK lens sill remain to be studied, their ability to control the development of myopia has gradually become the consensus [59,60]. These may help control the speed of development of myopia and are currently in common use in China. No one has found a way to stop or reverse the process, but there is a positive significance in controlling the number and severity of myopia [1].

## Conclusion

In summary, from this investigation, it could be concluded that the AL/CR ratio can explain the total variance in SE better than AL alone, but there may be other biometric parameters joined into the shaping of refraction except the AL/CR ratio. The effectiveness of the AL/CR ratio was statistically significantly better than UCVA for detecting myopia in children, and combining the two produced increased sensitivity without significantly decreasing specificity.
